# Teacher perspectives on the socio-ecological barriers and enablers to food and nutrition education in primary schools: a scoping review

**DOI:** 10.1017/S1368980024001812

**Published:** 2024-09-26

**Authors:** Emma K Esdaile, Lee Wharton, Helen Vidgen, Danielle Gallegos

**Affiliations:** 1 School of Nutrition and Exercise Sciences, Queensland University of Technology (QUT), Brisbane, QLD, Australia; 2 Centre for Childhood Nutrition Research (CCNR), Queensland University of Technology (QUT), Brisbane, QLD, Australia

**Keywords:** Food and nutrition education, Teacher perspectives, Self-efficacy, Policy, Cross-curriculum

## Abstract

**Objective:**

Schools are identified as a setting for food and nutrition education (FNE) in childhood. FNE is a key strategy to optimise child growth and development and impart life-long food skills. There is limited synthesis of the literature to understand the socio-ecological determinants of teachers and schools engaging in FNE.

**Design:**

For this scoping review, five databases (APA PsycInfo, ERIC, Medline, CINAHL and Scopus) were searched using the terms (and synonyms for) primary school teacher, self-efficacy and food and nutrition. A quality assessment using relevant Johanna Briggs tools was carried out for the included papers. Data were extracted using a modified socio-ecological model, and narrative themes were identified.

**Setting:**

Primary (elementary) schools in high-income countries.

**Participants:**

Primary-school teachers.

**Results:**

Forty-one papers were included in this review from ten countries (predominantly the USA). The narrative synthesis identified five themes that interact with teacher delivery of FNE. These were (i) perceived food and nutrition responsibilities of teachers, (ii) teacher beliefs and self-efficacy, (iii) opportunities to build teacher nutrition knowledge and self-efficacy, (iv) interpersonal contributors and (v) broader environmental, structural and policy contributors.

**Conclusions:**

Multiple strategies are needed to build the capacity of teachers to undertake FNE within primary school settings. These strategies include a focus on learner-centred education that will build teacher agency, school leadership, ensuring the health and well-being of teachers and providing initial teacher education as well as innovative professional development for cross-curriculum integration. Strategies drawing from each level of the socio-ecological framework will increase opportunities for capacity building.

Nutrition for optimal growth and development of children is a key feature for the health and well-being of children in the short-term and as they grow into adulthood^(1,2)^. Childhood is a critical window that establishes life-long eating habits and food preferences which continue to follow an established trajectory across the lifespan^(1–4)^. The environments in which children live, learn and play are key to providing access to information and education about nutrition and healthy eating as well as access to healthy food choices^(1,5)^. Internationally, the WHO and the United Nations FAO have identified schools as key to creating an enabling environment for children that promotes healthy eating and good nutrition^(6–8)^. In high-income countries, most children attend school for 5 days a week, for up to 30 h, meaning that schools are an opportunity for continuous, intensive contact with children and potentially their families and communities^(9,10)^. In response, the FAO has developed the *School-based food and nutrition education framework* to support agencies to develop, strengthen and support school food policies and programmes that have a collective impact on childhood nutrition, community development and local food systems^(11)^. The framework speaks to the importance of incorporating food and nutrition education (FNE) (defined as educational strategies and learning activities that are integrated in the curriculum) and healthy food provisioning across the entire school, specifically through the incorporation of FNE into regular school activities, the active involvement of parents, provision of fruit and vegetables within the classroom and ongoing integration of food and nutrition into curricula^(11)^. The framework includes activities from each element of the socio-ecological model (sem)^(12)^, in that it includes the individual, the household, the community and broader policy context. This scoping review is focused on the FNE aspect of the FAO framework, specifically the teacher and their capacity to deliver FNE in the classroom. It focused on primary (or elementary) schools as an environment where children (aged 5–11 years) are undertaking foundational learning including the development of life-long eating habits and food preferences.

School-based FNE programmes delivered in primary school settings have been shown to be effective in positively influencing children’s energy intakes, fruit and vegetable consumption and nutrition knowledge^(13,14)^. They are also linked to improved psychological and behavioural outcomes as well as academic performance^(15,16)^. While schools are recognised as ideal settings and nutrition education has been shown to be effective, the capacity of the school and its workforce to implement these interventions is often not considered^(15,17)^. Despite some evidence of teacher motivation^(18)^, a lack of resources, time and pedagogy knowledge as well as the impact of high-stakes assessment and curriculum have all been identified as limiting the ability of schools and teachers to actively embed FNE^(10,13,19,20)^. If schools are to deliver FNE then we need to understand the enablers and barriers that affect teacher’s self-efficacy related to the design and delivery of effective learning episodes on food and nutrition. This review sought to investigate factors impacting on the capacity of this workforce to deliver FNE. It took a socio-ecological perspective to explore the intra- and interpersonal, school, external and policy environmental factors that impact on the ability and likelihood of primary school teachers in high-income countries to deliver nutrition education.

## Methods

This scoping review was designed using the *Preferred Reporting Items for Systematic reviews and Meta-Analyses extension for Scoping Reviews (PRISMA-ScR) Checklist*^(21)^ and was registered on The Open Science Framework (9 November 2022) (https://doi.org/10.17605/OSF.IO/VAMJ4). The review was guided by the following research question: among teachers at primary schools in high-income countries, what are the attitudes, knowledge, beliefs, perceptions, confidence, capacity and self-efficacy relating to the teaching, delivery and management of school-based FNE?

Systematic searches of five databases (APA PsycInfo, ERIC, Medline, CINAHL and Scopus) were undertaken using the terms ‘primary/elementary school teacher’ AND ‘self efficacy’ AND ‘food’ OR ‘nutrition’ and a combination of synonyms of these terms (further search detail provided in see online supplementary material, Supplementary File 1). Database functionality was used to filter the results to include only peer-reviewed articles published in English from 2005 until May 2023. Results were downloaded to Endnote (Clarivate, USA) and duplicates removed. Eligibility screening of the results was undertaken against the criteria in Table 1.

**Table 1:** Inclusion criteria

Criterion	Description
Primary/elementary school teacher perspectives	Papers were included if they solely focussed on primary/elementary school teachers or if included in a larger cohort, results were reported separately
Food and nutrition education	Including FNE specifically or food and nutrition as part of lifestyle, obesity or health programmes/curricula. Papers that considered the impacts of school culture and government policy on teacher capacity to teach were also included
Country context	Papers were included if they were from a country that is a member of the Organisation for Economic Co-operation and Development (as predominantly democratic and supporting a free-market economy) and high-income as defined by the World Bank (https://data.worldbank.org/country/XD)
Peer-reviewed	Commentaries, conference abstracts and theses were excluded

A title screen was undertaken by one researcher (EE) with 20 % of the results checked by a second researcher. Results from this stage were uploaded to Covidence (Veritas Health Innovation, Vic, Australia) where two researchers (EE, DG) conducted an abstract then full-text review. Articles from the full-text review underwent forwards and backwards citation searches to identify additional articles for inclusion. A critical appraisal of all included studies was undertaken by two authors (EE, DG) using the Joanna Briggs Institute quality assessment tools for cross-sectional^(22)^, quasi-experimental^(23)^ and qualitative^(24)^ study designs. The Joanna Briggs Institute tools were used as they provide a coherent suite of tools for multiple study types.

The data charting process was iterative and collaborative. Two authors discussed the initial impressions of the papers and developed a framework to extract significant results from the included studies in Covidence, led by one author (EE) with 20 % cross-referenced by a second author (DG). Synthesis included a narrative interpretation of the quantitative data that was then integrated with the qualitative themes using a convergent integrated approach^(25)^.

**Fig. 1:**
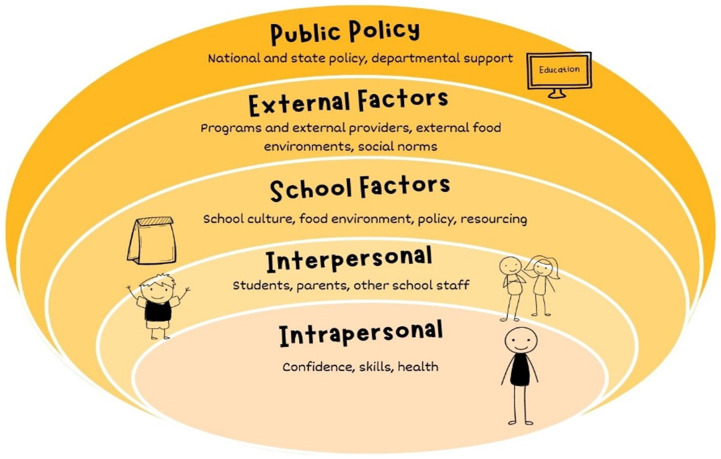
Ecological model for nutrition education in schools adapted from^(26,27)^

The socio-ecological model is used extensively as a conceptual framework to understand the multiple interacting determinants that impact on child health and development. It posits that children are at the centre of complex interactions between intrapersonal (individual characteristics), interpersonal (interactions and relationships between people), institutional (interactions with organisations (e.g. the school as an institution), community (socio-cultural norms, external agents) and broad macro policy factors^(12,26,27)^. The synthesis of results was interpreted using the ecological model adapted to nutrition education in schools by two authors (EE, DG) articulated in Fig. 1.

## Results

**Fig. 2:**
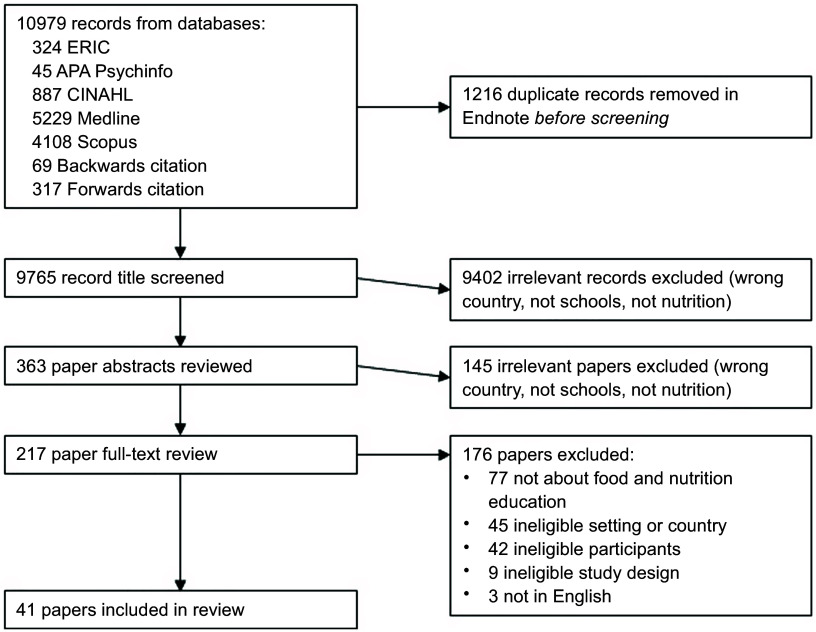
PRISMA diagram – identification of studies included in the scoping review

In total, forty-one studies were analysed for the scoping review (see Fig. 2). These included nine quasi-experimental (six high and three medium confidence of quality), twenty cross-sectional (eleven high, two medium, four low confidence and three poor quality), eleven qualitative studies (four high, three medium, one low confidence and three poor quality) and one mixed methods (high quality for qualitative, poor quality for cross-sectional) studies. Detailed quality assessment can be found in see online supplementary material, Supplementary File 2.

Most studies were from the USA (*n* 27), with four Australian studies, two studies each from Greece and Cyprus and one each from Chile, England, Finland, Korea, Norway and Sweden. Table 2 identifies which aspects of our modified socio-ecological model (Fig. 1) each study reported on. While all studies reported on intrapersonal factors (*n* 41), just under two-thirds of studies reported on school factors (*n* 26), about half reported on interpersonal (*n* 18) and external factors (*n* 19) and less than one in five reported on public policy factors (*n* 8). Further details of study design, methods, country, teacher characteristics, theoretical framework and a summary of findings can be found in see online supplementary material, Supplementary File 3.

**Table 2:** Included studies by area of socio-ecological model and study type (by first author surname, year, reference)

	Quasi-experimental	Cross-sectional	Qualitative	Mixed Methods
Intrapersonal Total = 41	Arcan 2013^(28)^; Fahlman 2013^(29)^; Hawkins 2021^(30)^; Katsagoni 2019^(31)^; Kulinna 2011^(32)^; Laitinen 2022^(33)^; Myers 2018^(34)^; Ritter-Gooder 2019^(35)^; Stage 2016^(36)^	Bae 2021^(37)^; Coccia 2020^(38)^; DeVleiger 2019^(39)^; Findholt 2016^(40)^; Graham 2005^(41)^; Hamilton 2021^(42)^; Hammerschmidt 2011^(43)^; Harris 2021^(44)^; Hart 2020^(45)^; Henry 2010^(46)^; Jones 2015^(47)^; Kinsler 2012^(48)^; Lambert 2006^(49)^; Lambert 2010^(50)^; Lambert 2016^(51)^; Metos 2019^(52)^; Perikkou 2015^(53)^; Prescott 2018^(54)^; Rafiroiu 2005^(55)^; Rossiter 2007^(56)^	Aydin 2021^(57)^; Aydin 2022^(58)^; Beinert 2021^(59)^; Berggren 2021^(60)^; Bergling 2021^(61)^; Gray 2016^(62)^; Hall 2016^(63)^; Koutsaki 2022^(64)^; Maliotou 2022^(65)^; Prelip 2006^(66)^; Vio 2018^(67)^	Bergling 2022^(68)^
Subtotals	9	20	11	1
Interpersonal Total = 18	Hawkins 2021^(30)^; Laitinen 2022^(33)^; Myers 2018^(34)^; Ritter-Gooder 2019^(35)^; Stage 2016^(36)^	Coccia 2020^(38)^; Findholt 2016^(40)^; Graham 2005^(41)^; Henry 2010^(46)^; Lambert 2006^(49)^; Lambert 2016^(51)^; Metos 2019^(52)^	Aydin 2021^(57)^; Beinert 2021^(59)^; Berggren 2021^(60)^; Gray 2016^(62)^; Hall 2016^(63)^; Koutsaki 2022^(64)^	
Subtotals	5	7	6	0
School factors Total = 26	Arcan 2013^(28)^; Katsagoni 2019^(31)^; Laitinen 2022^(33)^	Bae 2021^(37)^; Coccia 2020^(38)^; DeVleiger 2019^(39)^; Findholt 2016^(40)^; Graham 2005^(41)^; Hamilton 2021^(42)^; Hammerschmidt 2011^(43)^; Hart 2020^(45)^; Henry 2010^(46)^; Jones 2015^(47)^; Lambert 2006^(49)^; Lambert 2010^(50)^; Metos 2019^(52)^	Aydin 2021^(57)^; Aydin 2022^(58)^; Beinert 2021^(59)^; Berggren 2021^(60)^; Bergling 2021^(61)^; Gray 2016^(62)^; Hall 2016^(63)^; Koutsaki 2022^(64)^; Maliotou 2022^(65)^; Prelip 2006^(66)^	
Subtotals	3	13	10	0
External Factors Total = 19	Arcan 2013^(28)^; Fahlman 2013^(29)^; Hawkins 2021^(30)^; Katsagoni 2019^(31)^; Kulinna 2011^(32)^; Laitinen 2022^(33)^; Stage 2016^(36)^	Coccia 2020^(38)^; DeVleiger 2019^(39)^; Graham 2005^(40)^; Hammerschmidt 2011^(43)^; Harris 2021^(44)^; Henry 2010^(46)^; Lambert 2006^(49)^; Metos 2019^(52)^	Aydin 2022^(58)^; Maliotou 2022^(65)^; Vio 2018^(67)^	Bergling 2022^(68)^
Subtotals	7	8	3	1
Public policy Total = 8		DeVleiger 2019^(39)^; Findholt 2016^(40)^; Graham 2005^(41)^; Hamilton 2021^(42)^; Hart 2020^(45)^; Henry 2010^(46)^; Lambert 2006^(49)^; Lambert 2010^(50)^		
Subtotals	0	8	0	0

**Fig. 3:**
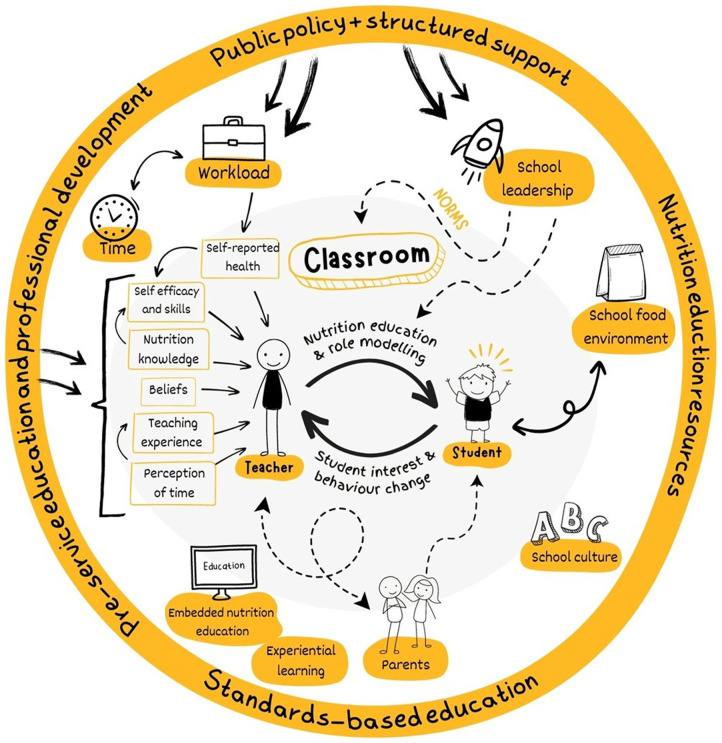
The identified components and interactions of the teaching ecosystem for food and nutrition education

Many of the included studies were focussed on FNE for the purpose of preventing or reducing childhood obesity and identified schools as a key setting for behaviour change interventions. Due to the diversity of papers and in keeping with a scoping review, we have identified five themes which align with the socio-ecological model. These were (i) perceived food and nutrition responsibilities of teachers, (ii) teacher beliefs and self-efficacy, (iii) opportunities to build teacher nutrition knowledge and self-efficacy, (iv) interpersonal contributors and (v) broader environmental, structural and policy contributors. The results were mind-mapped and are schematically represented in Fig. 3 (arrows are not intended to imply effect size).

### Perceived food and nutrition responsibilities of teachers

Teachers saw themselves fulfilling two main roles related to FNE in schools: educators and role models^(57,63,65)^.

#### Educators

In the identified studies, a majority of teachers from USA (79 %)^(36)^, Greece (87 %)^(31)^ and Finland (89 %)^(33)^ believed it was part of a teachers’ role to provide FNE, which was reiterated in qualitative studies^(58,61)^. Between 64 and 76 % of teachers reported delivering FNE as a standalone subject or integrated with other subjects, such as mathematics, science and humanities^(44,47,49,66)^. One-third of teachers (*N* 221, 30 %) reported including nutrition competencies in their lesson plans^(50)^ and half (*N* 482, 53 %) reported the use of formal lesson plans^(49)^. There was limited reporting of the volume of teaching time dedicated to FNE; however, three studies found between half^(44,45)^ and two-thirds^(49)^ of teachers taught 1–10 h annually, and another study found that about half of teachers taught 1–5 h annually^(52)^. One study found that the number of FNE hours was predicted by teachers’ beliefs regarding their impact on students and their self-efficacy to deliver FNE^(52)^.

#### Role models

Teachers recognised their potential influence as role models^(52,57,64)^. Most teachers, in one study, believed they could make a difference to student health behaviour (*N* 628, 81 %)^(52)^, and in another that their own eating behaviours influenced student eating behaviour (*N* 87, 71 %)^(40)^. Healthy eating role modelling was typically defined as teacher consumption of healthy foods and water during class time^(40,42)^. Teachers reported healthy eating role modelling about once a week while concurrently reporting unhealthy role modelling about once a fortnight^(42)^. Another study reported high consumption of water (*N* 87, 87 %) and ‘low fat snacks’ (88 %) in conjunction with moderate consumption of energy dense-nutrient poor snacks (78 %)^(40)^. Between one-third (*N* 75, 66 %)^(28)^ and nearly one-half (43 %) reported consuming sugar-sweetened beverages in class^(40)^.

Self-reported poor personal health also impacted on teachers’ ability to act as effective role models^(38,42)^. A study including pre-service teachers (individuals enrolled in a teacher education programme) (*N* 90) found that those with lower self-reported personal health and lower BMI had a higher likelihood of using confectionary as a reward (as a proxy for unsuitable role modelling). This association potentially reflects that teachers with weight issues may be more aware of the relationship between weight gain and food rewards^(38)^. Teachers with higher self-reported personal health were more likely to report healthy modelling and were less likely to engage in unhealthy classroom food practices^(42)^. This was only possible if teachers were permitted to eat with students and if the school food environment supported healthy eating messages^(60)^. Being a role model, however, needs to be balanced with the rights of teachers to privacy and downtime, essential for the management of teacher stress^(60)^. This potentially limited the opportunities for school meals to be opportunities for role modelling.

### Teachers’ beliefs and self-efficacy

A range of factors were identified as influencing role modelling and the teaching of nutrition including sex, age and years of experience^(37,42,46,48,53)^. Personal factors in Korea (age (being older), sex (being a woman)) were the only socio-demographics that remained significant for FNE after considering school type, school culture and health literacy^(37)^. More years of teaching experience increased confidence/self-efficacy to implement (plan and deliver) FNE^(46,48,53)^. Conversely, fewer years of teaching experience was also associated with higher endorsement of healthy role modelling practices^(42)^.

#### Beliefs regarding the importance of nutrition

Across multiple studies, there was consistently high agreement (92–97 %) that nutrition and the foods students eat impacted on learning and on current^(46)^ and future health^(38,40,46,52,58)^. If teachers believed nutrition was as important as other subjects, it was prioritised^(63)^. However, prioritisation was influenced by teachers’ personal beliefs and values related to the importance of nutrition and health^(61,68)^. Teachers across several studies agreed FNE should be included in their students’ curriculum^(49,64)^, should be compulsory^(64)^ and taught across all age groups^(39,55)^. In an Australian study (*N* 97), teachers unanimously reported that educating students about the benefits of consuming vegetables was valuable^(35)^. Despite these consistent beliefs about the importance of nutrition for their students, one study reported that three-quarters (*N* 69, 74 %) of teachers felt their students did not receive enough FNE^(43)^.

#### Self-efficacy

Self-efficacy to teach FNE was moderate^(30,46,49,50)^. Higher self-efficacy was found to help overcome perceived barriers to FNE^(53)^ and was predictive of the number of FNE hours taught^(52)^. Once teachers felt they had effectively taught health content (including FNE), they were more likely to embed it into their practice^(32)^.

Teachers with higher self-reported personal health had higher FNE self-efficacy^(38)^ and were more likely to deliver self-led or supported FNE lessons^(53)^. However, another study found teacher’s personal health, attitudes and beliefs had only a modest influence on classroom practices^(52)^. Studies focused on pre-service teachers reported less than half indicated good personal health, satisfaction with their own eating habits^(38)^ and higher likelihood of eating energy dense-nutrient poor foods^(56)^. Health was conceptualised as weight status in some studies, although no significant relationship was found between weight status and teacher’s confidence with FNE^(38,42,54)^. Another study found that teachers’ personal health was more relevant than nutrition knowledge in influencing food-related practices and modelling in classrooms^(42)^.

#### Nutrition knowledge and food skills

Nutrition knowledge was regarded as poor to fair among teachers and pre-service teachers^(31,38,47,55)^. In one study, a quarter (*N* 1094, 24 %) of teachers believed they had the nutrition knowledge to provide FNE to their students^(31)^. In that study, higher nutrition knowledge was positively associated with teacher attitudes towards role modelling, their beliefs about the importance of nutrition and their age but not their years of teaching experience nor position as specialist (health) teachers^(31)^. While in one study, nutrition knowledge did not appear to be associated with FNE self-efficacy^(48)^, in two other studies, teachers cited limitations in their nutrition knowledge as an intrapersonal barrier to FNE^(47,64)^.

### Building teacher nutrition knowledge and self-efficacy

#### Initial teacher education

Training provided during initial teacher education was identified as potentially improving teacher attitudes towards FNE, nutrition knowledge and FNE self-efficacy. Most pre-service teachers in one study in the USA reported they had little to no confidence in their initial teacher education training to prepare them to teach nutrition concepts effectively or to answer students’ nutrition-related questions^(38)^. Participation in a health methods class among pre-service teachers (USA), with quality skills-based instruction, effectively increased self-efficacy and outcome expectancy for health education (including nutrition) post-intervention^(29)^.

#### Professional development

In several studies across the USA, Australia and Greece teachers reported inadequate in-service training to incorporate nutrition into curricula^(50,58,64)^. Participation in FNE professional development interventions had mixed outcomes for teachers. In some studies (Australia, Cyrpus, USA), confidence to teach FNE improved with training and the provision of resources,^(35,53)^ which could remain stable over time^(35)^. Teachers in the USA undertaking some form of professional development, tended to report a greater understanding of concepts^(36)^ and improved classroom food practices^(28)^. Engagement in professional development also led to teachers being more receptive to FNE resources, school food and nutrition guidelines and the implementation of FNE in their classrooms^(46)^. However, other professional development interventions (one-day, e-learning) showed no improvements in confidence to teach FNE^(30)^, teacher perceptions of their influence on student nutrition knowledge or attitudes^(36)^ or changes to their intentions to teach FNE to their students^(31)^. The length of time devoted to professional development varied from intensive 1–2-day sessions^(28,36)^ to less intensive sessions spread over time^(40)^.

#### Resources

Access to easy-to-use teaching resources increased teacher confidence and knowledge^(57,63)^. Teachers valued resources that were geared towards being engaging for students, especially those with interactive elements including real food experiences^(31,39,59)^ or enjoyable activities and games^(34,35,67)^. Across multiple studies between a quarter and a third of teachers (25–33 %) consistently reported inadequate access to (or ability to identify) quality resources or the food/nutrition information they wanted to deliver FNE^(31,39,46,48,53)^. Teachers widely supported the provision of resources for FNE, including lesson plans, curriculum resources, information about how to integrate nutrition with other learning areas and materials for engagement with parents^(34,41,44,47)^.

There were indications that teachers, while appreciating lesson plans and outlines, also wanted them to be adaptable and flexible^(66)^. This flexibility allowed teachers the agency to apply a strengths-based approach to determining the content they delivered^(61,68)^ and in doing so influenced curriculum through adaptation in their individual classrooms^(63)^. Teachers also wanted to protect their autonomy to decide what foods were allowed in their classrooms^(51)^, despite mostly low endorsement of unhealthy classroom food practices^(42)^.

The sources of materials mattered to teachers. One study noted that sources of FNE materials were ad hoc and included websites, other teachers and sponsored education materials^(47)^. A lack of awareness of availability (and accessibility) of appropriate nutrition resources was identified as a key barrier to FNE^(47)^. Teachers reported high use of online information such as websites and games as resource materials to teach nutrition^(39)^. Another study reported that teachers moderately agreed on the importance of access to FNE materials from government agencies, including reliable information for their own knowledge^(46)^. Programmes such as *Crunch & Sip* (an in-class fruit, vegetable and water consumption programme, in Australia) that provided initial and ongoing materials were highly regarded by teachers^(34)^.

### Interpersonal contributors

#### Within-school networks and school leadership

Principals were identified as key agents influencing teacher beliefs, attitudes and behaviours and therefore their ability to act as food and nutrition educators. Principal support was also considered key for the integration of food and nutrition into curricula (see also the section below on school culture)^(51,58,65)^. Additionally, teachers’ beliefs about the acceptability of the use of energy dense-nutrient poor foods were found to be highly influenced by their perceptions about how others (principals, parents and other teachers) viewed their use^(51)^. One study identified that less than half (48 %) of teachers reported they had support from, or opportunity to collaborate with (39 %), other teachers or staff at school or outside nutrition expertise^(49)^.

#### What students get out of food and nutrition education matters

The top three reasons teachers gave for teaching FNE included its importance as a topic, their enjoyment of teaching it and their student’s interest^(44)^. Teachers’ enjoyment of FNE was enhanced if students had positive attitudes towards the topic^(63)^. In several studies, teachers indicated students were more engaged in FNE which included interactive components^(58,59,63,67)^ and experiential learning (typically cooking)^(54,64)^.

Views about how much interest students would take in FNE varied. One study reported the intention to teach FNE was strongly associated with teacher beliefs about outcome expectations for student behaviours (i.e. beyond food and nutrition knowledge)^(29)^. A study reported teachers believed that more hours of FNE teaching would have the biggest impact on student knowledge, attitudes and behaviours^(36)^. In another study, teachers reported enhanced FNE enjoyment if they perceived a change in student dietary behaviour^(61)^. However, other studies reported the opposite, revealing teachers did not believe that students would change their behaviour because of the information provided via a FNE intervention^(31)^, or that more time spent on FNE would have greater impacts on student knowledge, attitudes and beliefs about nutrition^(35)^.

#### Parents and the connection to home

Teachers believe that parents play an important role in establishing the dietary patterns of children and reinforcing healthy eating^(64)^. In one study, 25 % of teachers believed it was the responsibility of parents to teach children about nutrition^(39)^. Teachers were identified as the link between parents, students and the school environment with the potential to reach parents and provide them with support and resources to enable them to feed their children healthily^(58,64)^. Teachers in Greece believed there was an opportunity for students to apply the knowledge they learnt in the classroom to the home environment^(64)^. Two studies reported teachers’ interest in materials and resources to share with parents around nutrition education^(34,44)^ and for developing closer links with parents^(65)^.

### Broader environmental, structural and policy contributors

#### School culture

One study investigating the impact of school culture found it explained significant variance in teacher health-promoting behaviours, with positive school culture predicting health-promoting behaviours, interpersonal relations and stress management^(37)^. In many studies, teachers made the link between FNE, the school food environment and student food-related behaviours^(28,40,43,45)^. Teachers were cognisant that the school environment including, where provided, school meals was often the only exposure to healthy eating some children received^(63)^. Overall teachers believed that there should be some parameters about the quality of foods provided at school and were generally supportive of measures to improve the nutritional quality of school-provided meals and to remove or minimise energy dense-nutrient poor foods and beverages from vending machines, canteens, within school fundraising activities and the removal of marketing or advertising of these products on school grounds^(28,38,40,46,52,56)^.

Teachers generally reported having below-average support from the school to provide FNE in the classroom, such as appropriate materials (resources, books, curriculum/syllabus), equipment, funding and training (in-service)^(34,39,45–47,49,50,52)^. In England, strong school leadership was identified as a key factor for the successful implementation of whole school policy and FNE^(45)^. In this English study, only about a half (47 %) of primary schools had school governing bodies actively supporting and monitoring school food practices with senior leadership oversight of school food environments and FNE^(45)^.

#### Lack of time

Time was identified as a critical factor for the implementation of formal and informal FNE in the classroom^(45,58–61,63–68)^, some citing an already overcrowded curriculum^(58,65)^, or higher academic priorities (reading, maths, language) and associated high-stakes assessment^(34,39,41,43,44,47,49,50)^. Finding time in the curriculum to be able to engage both theory and practice was difficult, acknowledging that more time would promote deeper learning^(59)^. However, two studies reported that the perception of time and workload barriers (especially the burden of implementation in addition to other responsibilities) diminished when self-efficacy and familiarity with FNE increased^(61,68)^ despite no net change to curriculum or assessment overall.

#### Policy

Few studies directly explored the role of wider policy in supporting teachers to teach FNE. In the USA, schools are required to develop a School Wellness Policy aimed at student wellness that includes FNE. There is, however, flexibility within school districts to interpret what represents student wellness locally^(40,42)^. While one study found more than half of schools had integrated FNE via this policy mechanism^(43)^, another study found that teacher awareness and confidence to implement their school wellness policy tended to be low^(46)^. Other studies reported that around one-third (36 %) of teachers were unaware if their school had a food/nutrition policy^(8)^ or had participated in any FNE education programmes^(52)^. However, exploration of urban and suburban schools with established School Wellness Policies found over time teachers were less likely to endorse the use of food in the classroom^(42)^. Conversely, another study found there was little change in rural teacher food-related behaviours over the 10 years since the School Wellness Policy legislation had been passed^(40)^.

#### Embedding food and nutrition education

The three most common ways FNE was integrated into schools was through a school wellness or food and nutrition policy, embedded within FNE-specific health curriculum or other curricula, or by using external providers^(43)^. School meals where offered may be an opportunity for embedding FNE. However, the studies included in this review found no pertinent examples of this integration. Teachers consistently reported moderate agreement for the need to have a written school food and nutrition policy or guidelines to support FNE^(28,40,46)^. About one-third of teachers reported they believed they were given opportunities to influence school food/wellness policies including how to incorporate nutrition competencies into lesson plans^(28,50)^. The focus and the rigidity of the curriculum concentrating on what are considered core academic subjects (and high-stakes assessment) was identified as another barrier^(63,65)^. The pressures for teachers in a standards-based education system were identified as a barrier to some FNE activities (e.g. school gardens); however, the integration of nutrition into core academic subjects was identified as overcoming this^(41)^.

#### External providers of food and nutrition education

Access to and use of external providers for FNE in classrooms or to integrate FNE into schools were reported by between one-third and one-half of teachers across several studies^(43,49,50,52)^. One study identified moderate agreement of the importance of external providers being nutrition experts^(46)^. Another identified that schools tended to utilise existing contacts as external providers to deliver FNE (mostly parents, college students or other teachers with far fewer dietitians, school nurse or food staff)^(52)^. A qualitative study in Greece identified the need for a health professional in schools to support the nutrition environment^(64)^. Another advocated for a FNE coordinator position at the district (regional) level, to assist schools in developing FNE syllabus and facilitating lessons between schools^(50)^.

## Discussion

This review has identified that influencing the capacity of teachers to engage in nutrition education in primary schools is a complex interaction between broad education policy, leadership within schools, allocation of and access to resources and individual teacher factors. Most papers focussed on intra- and interpersonal factors with very few interrogating the influence of high-level policy. Focussing on more upstream factors will inevitably lead to addressing those issues for individual teachers effectively optimising the integration of FNE in primary schools.

One of the key upstream factors that will influence FNE in primary schools is a change in school culture. School culture is impacted by broad education policy as well as local school leadership (predominantly led by school principals)^(69)^. Teachers’ perceptions of the organisational structure within schools is postulated to impact on their experiences of burnout, time pressures, job satisfaction and self-efficacy^(70)^. A learning goal structure that focusses on individual student performance, and on safe and inspiring learning environments was associated with stronger self-efficacy and improved job satisfaction. A learning focus optimises teacher agency and brings to the forefront the skills of teachers in assessing individual student needs and adjusting accordingly. Conversely, a performance goal structure focuses on student performance and achievements and tends to be associated with increased experience of time pressures and burnout^(70,71)^. A performance goal structure has been the feature of neoliberal education policy in the UK, USA and Australia and has as its central features a focus on accountability: competition (between schools, teachers and students) and reporting scales^(20)^. The goal structure of a school is set by broader policy and by the school leadership^(72)^. The findings of this review suggest that teacher attitudes and food-related behaviours and their motivation to integrate FNE can change over a long period of time in response to national policy and/or policies at the school district level if they have appropriate structural support between the national-district-school nexus.

High-level policy that acknowledges and promotes the importance of food and nutrition is key to student learning. Strong school leadership to influence classroom food practices and whole-of-school cultural change is needed to embed normative practices and support teacher role modelling. Curriculum planning mechanisms are ideal for developing such FNE practices with local (community-relevant) contexts in mind. FNE in schools is optimised through facilitating cross-curricular integration of nutrition, a supportive school food environment (including promotion and curriculum integration of school meals and gardens)^(73,74)^ and the integration of experiential learning [policy] while at the same time promoting teacher agency^(19,75)^. Teacher agency has been widely contested but is broadly accepted as the degree to which teachers, as individuals but also as a community of professionals feel they have the power to act, make decisions and take a stance on approaches that affect their work and their identities^(76,77)^. The current predominant approach based on standardised-based policies, detailed national curriculum documents and national assessment protocols has constrained agency and has threatened the professional identities of teachers^(78)^. Despite these constraints one of the key features of agency is developing skills and competencies including the development of self-efficacy and capacity to negotiate between the constraints of policy and the needs of their learners^(79)^. The review highlights the need for a ‘bottom-up’ approach, acknowledging teachers as key agents in the transformation process of contextualising curriculum and integrating teaching and learning episodes, in this case that are focussed on FNE^(80,81)^.

There is growing international support for the integration of 21st century skills and competencies into curricula and this would include FNE as a life skill. Amid determining what 21st century skills and competencies might comprise, practicing teachers have identified an overcrowded curriculum and professional pressure to address national standards and accreditation values as barriers hindering the implementation of these skills and competencies^(82,83)^. At a more fundamental level, practicing teachers are unsure about the design, implementation and assessment of these ‘additional’ capabilities^(84)^. Integration of FNE with other curricula areas could be a means of overcoming the barriers expressed by teachers^(83,85–87)^. However, a performance focus and the siloing of key learning areas means that the integration of curriculum remains relatively unexplored. This can be partially overcome by aligning FNE content across other subjects considered more academically significant with high-stakes assessment.

Schools, heads of curriculum and teachers need to be given appropriate access to resources to ensure that integrating FNE reduces rather than increases workload. These resources are not necessarily lesson plans that limit teacher agency but rather resources that enable teachers to creatively adjust their teaching processes in a dynamic ongoing way. This review has also found that an increase in teacher self-efficacy and enhancement of their own health and well-being reduces perception of workload burden when adding FNE as part of their role. While teacher self-efficacy is optimised via the school structure and broader policy, the perception of lack of time and the workload associated with the burden of implementation of FNE that is in addition to their other responsibilities diminishes when self-efficacy and familiarity increase. The findings in this review indicated that providing professional development that is inclusive of self-efficacy principles is creative in how FNE is delivered across curriculum areas and that optimise teacher agency will be the most effective^(88)^.

It is increasingly acknowledged that teaching is a highly complex, stressful and demanding role^(89,90)^. In everyday practice, teachers encounter a range of challenges including but not limited to responding to varied student’s needs, navigating interpersonal relationships (with students, parents, other staff), managing expectations of all stakeholders, time pressures related to the demands of standards-based education and policy and work-life balance. With schools assuming a higher load associated with student’s psychosocial, emotional and physical well-being, this is increasing the burden on schools and teachers. If schools are to be sites that promote and teach health and well-being for its students, then the health and well-being of its teachers needs to be protected. Programmes to improve teacher health may improve their health behaviours and practices in schools and their motivation and self-efficacy to role model and teach food and nutrition^(70)^.

This review had three key limitations. Firstly, the papers included are heterogenous and many are small studies within localised contexts that may not be generalisable. This was overcome in part by applying an ecological lens to synthesise the data more broadly. Secondly, many of the papers emerged from school environments in which school meals are provided and the delivery of FNE in this context is very different to those where students bring food from home. Finally, this review was limited to primary/elementary school contexts and did not include middle or high school/secondary school, where eating behaviours and food literacy are still forming and where teachers can still be influential.

Based on this review, key recommendations for moving forward in building FNE into primary schools include as follows:

1. A continued focus on education reform to be more learner-centred to promote teacher agency;
2. Embedding FNE skills into initial teacher education for the next generation of teachers;
3. Ensuring that principals and school leadership teams understand the role and importance of FNE for student outcomes;
4. Adaptable and flexible integration of FNE into curriculum planning supported by school leadership;
5. Workplace environments that ensure and promote teacher health and well-being to support their capability to act as role models and
6. Providing resources to creatively integrate FNE across curricular areas to minimise workload, promote self-efficacy and build student life skills.

Finally, this review has demonstrated a complex interplay of factors that support or hinder the integration of FNE in primary school settings. These include interpersonal and intrapersonal teacher factors through to broader upstream school culture and policy influences. Taking a socio-ecological approach and working across systems will potentially enhance the capacity of schools to make FNE core business.

## Supporting information

Esdaile et al. supplementary material 1 Esdaile et al. supplementary material

Esdaile et al. supplementary material 2 Esdaile et al. supplementary material

Esdaile et al. supplementary material 3 Esdaile et al. supplementary material

## Supplementary material

For supplementary material accompanying this paper, visit https://doi.org/10.1017/S1368980024001812
