# Supplementary material for: Teacher perspectives on the socio-ecological barriers and enablers to food and nutrition education in primary schools: a scoping review
Source: Public Health Nutr. 2024 Sep 26;27(1):e175. doi: 10.1017/S1368980024001812 (PMC11504532; doi:10.1017/S1368980024001812)
Supplement: Esdaile et al. supplementary material 1 — Esdaile et al. supplementary material [file S1368980024001812sup001.docx]

**Supplementary File 1. Scoping review search strategy**

**Research Question**

Among teachers at primary schools in high income countries, what are the attitudes, knowledge, beliefs, perceptions, confidence, capacity and self-efficacy relating to the teaching, delivery and management of school-based food and nutrition education?

**P** – population: primary or elementary school teachers

**I** – self-efficacy, attitudes, knowledge, motivations, perceptions and beliefs

**C** – n/a

**O** – ability to enact food and nutrition curricular, programs and strategies

**Databases**

1. EBSCO Host

APA PsycInfo

CINAHL

ERIC

Medline

1. Scopus (Elsevier)

**Inclusion Criteria**

- English language
- Primary Schools
- Peer-reviewed
- High income country
- Year: from 2005

**Exclusion Criteria**

- Theses, media, magazines, letters, books, editorials, conference, abstracts, study protocols
- Not in English
- Middle or High School (adolescents)
- Kindergarten or preschool
- Low and middle income countries (as per UNDP)
- Interventions that do not report on teacher’s perspectives/beliefs etc
- School meals that do not report on teacher’s perspectives etc, reporting on teachers perspectives of uptake
- Not related to food or nutrition e.g. Physical activity/sports focus/biotechnology
- Reports on satisfaction of training only
- Knowledge and attitudes of students
- Knowledge and attitudes of parents
- Policies only

Note: Informit in new format and not able to download searches easily omitted

**Database Search terms**

## ERIC

| S1 | DE "Elementary School Teachers" OR ((primary OR elementary) N5 (teacher OR educator)) |
| --- | --- |
| S2 | (DE "Elementary Education") OR (DE "Elementary Schools") OR (TI("primary school*" OR "elementary school*")) OR (AB ("primary school*" OR "elementary school*")) |
| S3 | S1 OR S2 |
| S4 | ((((((((((((((DE "Teacher Response") OR (DE "Teacher Effectiveness")) OR (DE "Teacher Attitudes")) OR (DE "Teacher Competencies")) OR (DE "Knowledge Base for Teaching")) OR (DE "Pedagogical Content Knowledge")) OR (DE "Teacher Role")) OR (DE "Teacher Participation")) OR (DE "Teacher Surveys")) OR (DE "Teaching Experience")) OR (DE "Teacher Behavior")) OR (DE "Teacher Responsibility")) OR (DE "Teacher Motivation")) OR (DE "Teacher Influence") OR (teacher* N3 ("self efficacy" OR self-efficacy OR confidence OR behavio#r OR capacity OR interest OR attitude* OR knowledge OR belief*))) OR (DE "Self Efficacy") |
| S5 | (((((((((DE "Food") AND (DE "Cooking Instruction" OR DE "Home Economics" OR DE "Food Service")) AND (DE "Nutrition" OR DE "Nutrition Instruction")) OR (DE "Foods Instruction")) OR (DE "Breakfast Programs")) OR (DE "Lunch Programs")) OR (DE "Gardening")) OR (DE "Dining Facilities")) OR (DE "Dietetics")) OR (DE "Eating Habits") OR food* OR nutrition* OR diet* OR eating OR garden* OR canteen OR tuckshop OR meal OR breakfast OR lunch OR snack OR “food literacy” OR “nutrition literacy” OR “food environment” OR “food skills” OR cooking OR “nutrition knowledge” |
| S6 | S3 AND S5 AND S6 |
|  | English language, peer reviewed, 2005 -2023 |

## CINAHL

| S1 | (MH "Teachers") OR "teachers" OR (MH "Faculty+") OR ((primary OR elementary) N5 (teacher OR educator) |
| --- | --- |
| S2 | MH "Schools, Elementary") OR "elementary education" |
| S3 | S1 OR S2 |
| S4 | (MH "Self-Efficacy") OR (MH "Knowledge+") OR (MH "Professional Knowledge+") OR (MH "Health Knowledge") OR (MH "Attitude+") OR (MH "Health Beliefs") OR (MH "Behavior+") OR (MH "Motivation+") OR (MH "Confidence") OR ((teacher*) N3( "self efficacy" OR "confidence" OR "capacity" OR "attitude" OR "belief" OR "behavior" OR "behaviour" OR "motivation")) |
| S5 | (MH "Food+") OR "food" OR (MH "Nutrition+") OR "nutrition" OR (MH "Nutrition Policy+") OR (MH "Breakfast") OR "breakfast" OR (MH "Lunch") OR "lunch" OR (MH "Diet+") OR "diet" OR (MH "Eating") OR "eating" OR "garden" OR (MH "Horticulture") OR (MH "Meals+") OR "meals" OR (MH "Meal Preparation+") OR (MH "Snacks") OR (MH "Menu Planning") OR "school meals" OR "canteen" OR "tuckshop" OR “food literacy” OR “nutrition literacy” OR “nutrition knowledge” OR cooking |
| S4 | S3 AND S4 AND S5 |
|  | English language, peer reviewed, 2005 -2023 |

## APA PsychInfo

| S1 | DE "Elementary School Teachers" OR ((primary OR elementary) N5 (teacher OR educator)) |
| --- | --- |
| S2 | (DE "Elementary Education") OR (DE "Elementary Schools") OR (TI("primary school*" OR "elementary school*")) OR (AB ("primary school*" OR "elementary school*")) |
| S3 | S1 OR S2 |
| S4 | ((((((((((((((DE "Teacher Response") OR (DE "Teacher Effectiveness")) OR (DE "Teacher Attitudes")) OR (DE "Teacher Competencies")) OR (DE "Knowledge Base for Teaching")) OR (DE "Pedagogical Content Knowledge")) OR (DE "Teacher Role")) OR (DE "Teacher Participation")) OR (DE "Teacher Surveys")) OR (DE "Teaching Experience")) OR (DE "Teacher Behavior")) OR (DE "Teacher Responsibility")) OR (DE "Teacher Motivation")) OR (DE "Teacher Influence") OR (teacher* N3 ("self efficacy" OR self-efficacy OR confidence OR behavio#r OR capacity OR interest OR attitude* OR knowledge OR belief*))) OR (DE "Self Efficacy") |
| S5 | (((((((((DE "Food") AND (DE "Cooking Instruction" OR DE "Home Economics" OR DE "Food Service")) AND (DE "Nutrition" OR DE "Nutrition Instruction")) OR (DE "Foods Instruction")) OR (DE "Breakfast Programs")) OR (DE "Lunch Programs")) OR (DE "Gardening")) OR (DE "Dining Facilities")) OR (DE "Dietetics")) OR (DE "Eating Habits") OR food* OR nutrition* OR diet* OR eating OR garden* OR canteen OR tuckshop OR meal OR breakfast OR lunch OR snack OR “food literacy” OR “nutrition literacy” OR “food environment” OR “food skills” OR cooking |
| S6 | S3 AND S5 AND S6 |
|  | English language, peer reviewed, 2005 -2023 |

## Medline

| S1 | (MH "School Teachers") OR (MH "Educational Personnel+") OR (MH "Schools+") OR ((primary OR elementary) N5(teachers OR educators) |
| --- | --- |
| S2 | (MH "Self Efficacy") OR (MH "Knowledge") OR (MH "Knowledge Bases+") OR (MH "Health Knowledge, Attitudes, Practice") OR (MH "Attitude+") OR (MH "Attitude to Health+") OR (MH "Behavior+") OR (MH "Health Behavior+") OR (MH "Motivation+") OR ((teacher*) N3( "self efficacy" OR "confidence" OR "capacity" OR "attitude" OR "belief" OR "behavior" OR "behaviour" OR "motivation")) |
| S3 | (MH "Food+") OR "food" OR (MH "Nutrition+") OR "nutrition" OR (MH "Nutrition Policy+") OR (MH "Breakfast") OR "breakfast" OR (MH "Lunch") OR "lunch" OR (MH "Diet+") OR "diet" OR (MH "Eating") OR "eating" OR "garden" OR (MH "Horticulture") OR (MH "Meals+") OR "meals" OR (MH "Meal Preparation+") OR (MH "Snacks") OR (MH "Menu Planning") OR "school meals" OR "canteen" OR "tuckshop" OR “food literacy” OR “nutrition literacy” OR “nutrition knowledge” |
| S4 | S1 AND S2 AND S3 |
|  | English language, peer reviewed, 2005 -2023 |

## Scopus (Elsevier)

| S1 | ALL(“primary school” OR “elementary school” OR ((primary OR elementary) W/3 (teacher OR educator))) |
| --- | --- |
| AND S2 | ALL((teacher OR educator) W/3("self efficacy" OR self-efficacy OR behaviour OR behavior OR knowledge OR belief OR motivation OR effectiveness OR attitude OR capacity)) |
| AND S3 | ALL(food OR nutrition OR "food literacy" OR "nutrition literacy" OR "nutrition knowledge" OR cooking OR meals OR breakfast OR lunch OR snack OR eating OR "food preparation" OR "food service" OR "food supply" OR "food skills" OR canteen OR tuckshop) |
|  | English, Journal, Article, 2005-2023 |
