# Supplementary material for: Teacher perspectives on the socio-ecological barriers and enablers to food and nutrition education in primary schools: a scoping review
Source: Public Health Nutr. 2024 Sep 26;27(1):e175. doi: 10.1017/S1368980024001812 (PMC11504532; doi:10.1017/S1368980024001812)
Supplement: Esdaile et al. supplementary material 2 — Esdaile et al. supplementary material [file S1368980024001812sup002.docx]

**Supplementary File 2:** Quality assessment of included studies

**Quasi-experimental studies (n=9)**

|  | **Joanna Briggs Institute (JBI) quality assessment of quasi-experimental questions*** | | | | | | | | |  |
| --- | --- | --- | --- | --- | --- | --- | --- | --- | --- | --- |
| **Study ID** | **1** | **2** | **3** | **4** | **5** | **6** | **7** | **8** | **9** | **Decision** |
| Arcan 2013[28] | Yes | No | Yes | Yes | Yes | N/A | Yes | Yes | N/A | High confidence |
| Fahlman 2013[29] | Yes | Yes | Yes | Yes | Yes | Yes | Yes | Yes | Yes | High confidence |
| Hawkins 2021[30] | Yes | Unclear | Yes | Yes | Yes | Unclear | Yes | Yes | Yes | High confidence |
| Katsagoni 2019[31] | Yes | Yes | Yes | Yes | Yes | Yes | Unclear | Yes | Yes | High confidence |
| Laitinen 2022[32] | Yes | Yes | Yes | Yes | Yes | Yes | Yes | Yes | Yes | High confidence |
| Kulinna 2011[33] | Yes | Yes | Unclear | No | Yes | Unclear | N/A | Yes | Yes | Medium confidence |
| Myers 2018[34] | Yes | Yes | Yes | No | Yes | No | Yes | Unclear | Yes | Medium confidence |
| Ritter-Gooder 2019 [35] | Yes | Yes | Unclear | No | Yes | Yes | Unclear | Yes | Yes | Medium confidence |
| Stage 2016[36] | Yes | Unclear | Unclear | Yes | Yes | Unclear | Yes | Yes | Yes | High confidence |

***Reference**: Tufanaru C, Munn Z, Aromataris E, Campbell J, Hopp L. Chapter 3: Systematic reviews of effectiveness. In: Aromataris E, Munn Z (Editors). JBI Manual for Evidence Synthesis. JBI, 2020. Available from <https://synthesismanual.jbi.global>

| **Key to JBI quality assessment questions for quasi-experimental studies** | |
| --- | --- |
| 1 | Is it clear in the study what is the “cause” and what is the “effect”? |
| 2 | Were the participants included in any comparisons similar? |
| 3 | Were the participants included in any comparisons receiving the same treatment/care, other than the exposure or intervention of interest |
| 4 | Was there a control group? |
| 5 | Were there multiple measurements of the outcome both pre and post the intervention? |
| 6 | Was follow up complete and if not, were there differences between groups in terms of their follow up adequately described and analysed? |
| 7 | Were the outcomes of participants included in any comparisons measured in the same way? |
| 8 | Were the outcomes measured in a reliable way? |
| 9 | Was appropriate statistical analysis used? |

**Cross-sectional studies (n=20)**

|  | **JBI quality assessment of cross-sectional studies questions*** | | | | | | | |  |
| --- | --- | --- | --- | --- | --- | --- | --- | --- | --- |
| **Study ID** | **1** | **2** | **3** | **4** | **5** | **6** | **7** | **8** | **Decision** |
| Bae 2021 [37] | No | Yes | Yes | Yes | Yes | Yes | Yes | Yes | High confidence |
| Coccia 2020 [38] | Yes | Unclear | Yes | Yes | Yes | No | Yes | Yes | High confidence |
| deVlieger 2019 [39] | Yes | Yes | N/A | Yes | N/A | N/A | N/A | Yes | Low confidence |
| Findholt 2016 [40] | Yes | Yes | Yes | Yes | No | No | Yes | Unclear | Medium confidence |
| Graham 2005 [41] | Yes | No | Yes | Unclear | No | No | Unclear | Yes | Poor quality |
| Hamilton 2021 [42] | Unclear | Yes | Yes | Yes | Yes | Yes | Yes | Yes | High confidence |
| Hammerschmidt 2011 [43] | Yes | No | N/A | Unclear | No | Unclear | Unclear | No | Poor quality |
| Harris 2021 [44] | Yes | Yes | Unclear | Unclear | Yes | No | N/A | Unclear | Low confidence |
| Hart 2020 [45] | Yes | Yes | Unclear | Unclear | Yes | No | Unclear | N/A | Low confidence |
| Henry 2010 [46] | Yes | Yes | Yes | Yes | Yes | Yes | Yes | Yes | High confidence |
| Jones 2015 [47] | Yes | Yes | Yes | Yes | Yes | Unclear | N/A | Yes | High confidence |
| Kinsler 2012 [48] | Yes | Yes | No | Yes | Yes | Yes | Yes | Yes | High confidence |
| Lambert 2006 [49] | No | No | Yes | Yes | No | No | Yes | Yes | Poor quality |
| Lambert 2010 [50] | Yes | Yes | Yes | Yes | Unclear | No | Yes | Yes | High confidence |
| Lambert 2016 [51] | Yes | Unclear | Yes | Yes | Unclear | No | Yes | Yes | Low confidence |
| Metos 2019 [52] | Yes | Yes | Yes | Yes | Yes | Unclear | Yes | Yes | High confidence |
| Perikkou 2015 [53] | Yes | Yes | Yes | Yes | Yes | Yes | Unclear | Yes | High confidence |
| Prescott 2018 [54] | Yes | Yes | Unclear | Yes | Yes | Yes | Unclear | Yes | Medium confidence |
| Rafiroiu 2005 [55] | Yes | Yes | Yes | Unclear | Yes | No | Yes | Yes | High confidence |
| Rossiter 2007 [56] | Unclear | No | Yes | Yes | Yes | Yes | Yes | Yes | High confidence |

***Reference**: Moola S, Munn Z, Tufanaru C, Aromataris E, Sears K, Sfetcu R, Currie M, Qureshi R, Mattis P, Lisy K, Mu P-F. Chapter 7: Systematic reviews of etiology and risk. In: Aromataris E, Munn Z (Editors). JBI Manual for Evidence Synthesis. JBI, 2020. Available from <https://synthesismanual.jbi.global>

| **Key to JBI quality assessment questions for cross-sectional studies** | |
| --- | --- |
| 1 | Were the criteria for inclusion in the sample clearly defined? |
| 2 | Were the study subjects and the setting described in detail? |
| 3 | Was the exposure measured in a valid and reliable way? |
| 4 | Were objective, standard criteria used for measurement of the condition? |
| 5 | Were confounding factors identified? |
| 6 | Were strategies to deal with confounding factors stated? |
| 7 | Were the outcomes measured in a valid and reliable way? |
| 8 | Was appropriate statistical analysis? |

**Qualitative studies (n=11)**

|  | **JBI quality assessment of qualitative studies questions*** | | | | | | | | | | |  | |
| --- | --- | --- | --- | --- | --- | --- | --- | --- | --- | --- | --- | --- | --- |
| **Study ID** | **1** | **2** | **3** | **4** | **5** | **6** | **7** | **8** | **9** | **10** | **Decision** | |  |
| Aydin 2021 [57] | Unclear | Yes | Yes | Yes | Yes | No | No | Yes | Yes | Yes | Medium confidence | |  |
| Aydin 2022 [58] | Yes | Yes | Yes | Yes | Yes | No | Yes | Yes | Yes | Yes | High confidence | |  |
| Beinert 2021 [59] | No | Yes | No | Yes | Yes | No | Unclear | Yes | Yes | Yes | Medium confidence | |  |
| Berggren 2021 [60] | No | No | No | Yes | Yes | No | No | Yes | Yes | Yes | Poor quality | |  |
| Bergling 2021 [61] | Yes | Yes | Yes | Yes | Yes | No | No | Yes | Yes | Yes | Medium confidence | |  |
| Gray 2016 [62] | Yes | Yes | Yes | Yes | Yes | No | No | Yes | Yes | Yes | High confidence | |  |
| Hall 2016 [63] | Yes | Yes | Yes | Yes | Yes | No | Yes | Yes | Yes | Yes | High confidence | |  |
| Koutsaki 2023 [64] | Unclear | Yes | Yes | Yes | Yes | No | Yes | Yes | Yes | Yes | High confidence | |  |
| Maliotou 2022 [65] | No | Yes | Yes | Yes | Yes | No | No | Yes | No | Yes | Low confidence | |  |
| Prelip 2006 [66] | No | No | Unclear | Unclear | Unclear | No | No | No | Yes | No | Poor quality | |  |
| Vio 2018 [67] | No | Unclear | Unclear | Unclear | Yes | Unclear | No | Yes | Yes | Yes | Poor quality | |  |

***Reference**: Lockwood C, Munn Z, Porritt K. Qualitative research synthesis: methodological guidance for systematic reviewers utilizing meta-aggregation. Int J Evid Based Healthc. 2015;13(3):179–187

| **Key to JBI quality assessment questions for qualitative studies** | |
| --- | --- |
| 1 | Is there congruity between the stated philosophical perspective and the research methodology? |
| 2 | Is there congruity between the research methodology and the research question or objectives? |
| 3 | Is there congruity between the research methodology and the methods used to collect data? |
| 4 | Is there congruity between the research methodology and the representation and analysis of data? |
| 5 | Is there congruity between the research methodology and the interpretation of results? |
| 6 | Is there a statement locating the researcher culturally or theoretically? |
| 7 | Is the influence of the researcher on the research, and vice- versa, addressed? |
| 8 | Are participants, and their voices, adequately represented? |
| 9 | Is the research ethical according to current criteria or, for recent studies, and is there evidence of ethical approval by an appropriate body? |
| 10 | Do the conclusions drawn in the research report flow from the analysis, or interpretation, of the data? |

**Mixed methods (n=1)**

Bergling 2022 [68]

|  | **JBI quality assessment of qualitative and cross-sectional studies questions*** | | | | | | | | | |  |
| --- | --- | --- | --- | --- | --- | --- | --- | --- | --- | --- | --- |
| **Qualitative** | **1** | **2** | **3** | **4** | **5** | **6** | **7** | **8** | **9** | **10** | **Decision** |
|  | Yes | Yes | Yes | Yes | Yes | Unclear | Unclear | Yes | Yes | Yes | High confidence |
| **Cross-sectional** | **1** | **2** | **3** | **4** | **5** | **6** | **7** | **8** |  |  |  |
|  | Unclear | Yes | Unclear | Unclear | Unclear | Unclear | Unclear | Unclear |  |  | Poor quality |

*Refer to JBI quality assessment question keys above
