# Supplementary material for: Teacher perspectives on the socio-ecological barriers and enablers to food and nutrition education in primary schools: a scoping review
Source: Public Health Nutr. 2024 Sep 26;27(1):e175. doi: 10.1017/S1368980024001812 (PMC11504532; doi:10.1017/S1368980024001812)
Supplement: Esdaile et al. supplementary material 3 — Esdaile et al. supplementary material [file S1368980024001812sup003.docx]

**Supplementary File 3, Table 1**: Findings from Quasi-experimental Papers

| **Author**  **Year**  **Quality^**  **Country** | **Method** | **Sample size**  **N** | **Participants**  **Elementary school teachers*** | | | | **Frameworks** | | **Main Findings** |
| --- | --- | --- | --- | --- | --- | --- | --- | --- | --- |
|  |  |  | **n (%)** | **Sex**  **F (%)** | **Grades** | **Years Teaching** | **Theoretical** | **Socio-ecological framework** |  |
| Arcan  2013 [28]  High  USA | Surveys – baseline + post-intervention | 75 | I = 43 (57)  C = 32 (43) | NS | K-1 | NS | NS | Intrapersonal  School  External | Statistically significant change for 4 items, intervention teachers:  less frequently used fast food (P=0.008) or candy (P=0.0005) as reward/treat; more disagreed with provision of fast food being offered at school (P=0.019) the sale of candy, nachos or fry bread (P=0.006) that it does not make sense to limit school food choices when student have autonomy outside of school (P=0.035) |
| Fahlman  2013 [29]  High  USA | Surveys – baseline + post-intervention | 513 | Preservice  I = 285 (56)  C = 228 (44) | I (54)  C (50) | NA | NA | TPB  SCT | Intrapersonal External | Nutrition education outcome expectancies, sig interaction between the 2 groups at each time, large effect size F= 444.1 P≤.001, η^2^=0.79. Participants in the health methods class had a significant increase in outcome expectations from pre to post and those post scores were in turn significantly higher than the post scores of the control group. |
| Hawkins  2021 [30]  High  USA | Surveys – baseline + post-intervention | 55 teachers  (1302 students) | 55 (100) | 90 | 1-5 | NA | SEM | Intrapersonal  Interpersonal  External | Among the 55 teachers that participated in PD (up to five sessions), attending more PD sessions resulted in more nutrition lessons implemented in the classroom (r=0.6, p<0.05) |
| Katsagoni  2019 [31]  High  Greece | Surveys – baseline + post-intervention | 1^0^+2^0^+ PE  B= 1094  I = 619 | B = 520 (48)  I = 310 (50) | B (75)  I (76) | K-12 | 14 | NS | Intrapersonal  School External | Kindergarten and elementary school teacher results not reported separately.  Good teacher nutrition knowledge was associated with attitudes towards being effective role model (p<0.001) and beliefs re: importance of nutrition (p=0.003) after adjustment for age, sex etc)  An e-learning intervention improved knowledge 65% to 89% (p<0.001) as did teacher’s barriers and beliefs (p<0.001) but not perceptions. |
| Kulinna  2011 [32]  Medium  USA | Surveys – baseline + after each workshop + post-intervention | 1^0^ + 2^0^  50  Native  American community | 17 (34) | 54  whole sample | NS | 8.9 yrs | SE | Intrapersonal  External | ANOVA for linearity between student outcome scores and teacher participation levels was not significant for food pyramid knowledge, possibly because this was already integrated into lessons. Evidence does support positive impact of PD on teacher SE unclear whether this was workshops or mentoring. |
| Laitinen  2022 [33]  High  Finland | Surveys – baseline + post-intervention | 130 | I = 82 (63)  C = 48 (37) | I = 79  C = 75 | NS | NS | Feasibility | Intrapersonal  Interpersonal  School  External | Compared to the control group teachers in the intervention group had a significant (p=0.0017) increase in “food education can affect pupil’s wellbeing” and significant increases in having sufficient supplies, materials and supports to implement food education (P<0.001) |
| Myers  2018 [34]  Medium  Australia | Surveys – baseline + post-intervention | 61 | B=61  Process = 39  Outcome =35 | NS | 1-2, 5-6 | NS | NS | Intrapersonal  Interpersonal | Pilot implementation of fruit break in classroom. Improvements in perceived knowledge of veg benefits (p=0.001) and confidence to education students on benefits (p=0.028). Barriers identified as crowded curriculum, facilities, limited family support |
| Ritter-Gooder  2019 [35]  Medium  USA | Surveys after curriculum delivery over 4 years | 356 | Year:  1 = 135  2 = 110  3 = 51  4 = 60 | NS | K-2 | NS | SCT | Intrapersonal  Interpersonal | Significant increases in confidence level of basic food preparation and food safety skills between year 1 and year 2, in year 4, significant decline in perceived NK and food safety skills Significant increases in teacher confidence levels from year to year in some items, but not all. |
| Stage  2016 [36]  High  USA | Surveys – baseline + post-intervention | 34 | I = 19 (56)  C = 15 (44) | I = 90  C = 93 | 4 | 16.8 yrs  I:17.8 yrs  C:15.3 yrs | SE | Intrapersonal  Interpersonal External | After controlling for baseline scores Efficacy Expectations improved in intervention group (F = 17.50; df =1; P <.001), Outcome Expectations also improved (F = 5.88; df = 1; P = .02 |

^Paper quality, see Supplementary File 2.

*Elementary and primary school are used depending on country context

**Abbreviations**: 1^0^ + M + 2^0^ = primary/elementary + middle school + secondary/high school; B = baseline; C = control; CFIR = Consolidated Framework for Implementation Research; D&I = dissemination and implementation; F/u = follow-up; HBM = Health Belief Model; I = intervention; INEP = Integrated Nutrition Education Program; K = Kindergarten; LNV = Low nutritive value; NA = not applicable; NE = nutrition education; NK = nutrition knowledge; NS = not specified; OCT = Organisational Change Theory; PD = professional development; PE = physical education; PHI = personal health index; PRISM = Practical Implementation Sustainability Model; RE-AIM = Reach, Effectiveness, Adoption, Implementation, and Maintenance; SCT = Social Cognitive Theory; SE = Self Efficacy (Bandura); SEM = Socio-Ecological Model; SFE = school food environment; SSB = sugar sweetened beverages; TPB = Theory of Planned Behaviour; TTM = Transtheoretical Model (five stages of change).

**Supplementary File 3, Table 2**: Findings from Cross-sectional Papers

| **Author**  **Year**  **Quality^**  **Country** | **Method** | **Sample size**  **N** | **Participants**  **Elementary school teachers*** | | | | **Frameworks** | | **Main Findings** |
| --- | --- | --- | --- | --- | --- | --- | --- | --- | --- |
|  |  |  | **n (%)** | **Sex**  **F (%)** | **Grades** | **Years Teaching** | **Theoretical** | **Socio-ecological framework** |  |
| Bae  2021 [37]  High  Korea | Survey | 1^0^+M+2^0^  474 | 200 (42) | 78  whole sample | K-12 | NS | Sorensen integrated model of health literacy | Intrapersonal  School | Health literacy was a significant predictor in all HPLP-II domains (P<.001). School type influenced nutrition (= 0.10, P= .039) and stress management (= 0.12, P=.008), school culture predicted interpersonal relations (= 0.14, P<.001) and stress management (= 0.12, P= .005). |
| Coccia  2020 [38]  High  USA | Survey | Pre-service teachers  94 | 94 | 93 | NA | NA | SCT  TPB | Intrapersonal  Interpersonal  School  External | Personal health index was correlated to self-efficacy β =.45 (P<.01), nutrition knowledge β=-.31 (P<.01), beliefs about selling snacks β=-.29 (P<.01), use of candy as a reward β= -.25 (P<0.05); nutrition knowledge was correlated to beliefs about selling snacks β= -.32 (P<.01), Use of candy as a reward was correlated with beliefs about selling snacks β=.35 (P<.01) (Beta numbers)  The variable with the highest total effects influencing use of candy as a reward was pre-service teachers’ personal health index, followed by BMI and their belief that snacks should be sold at school. The model accounted for 21% of the observed variance in pre-service teachers’ use of candy as a reward. |
| DeVleiger  2019 [39]  Low  Australia | Survey | 33 | 33 | 85 | Grades 1-6 | 33%>16 yrs | NS | Intrapersonal  School  External  Public Policy | Descriptives. On average, the participants felt that the nutrition education materials are not up‐to‐date or sufficient (M= 3.58, SD 1.2). The mean interest in nutrition among the teachers in the study a sample was 1.81 (SD 0.74) (on a scale of 1 = very interested to 6 = not at all interested). On a scale of 1 to 7, where 1 is extremely positive and 7 extremely negative, the teachers scored a mean of 2.39 (SD 1.30) when asked about how they feel about integrating nutrition and health education into the math curriculum. 78.8%, 24.2% and 33% of teachers believed that “a lack of time”, not knowing enough about nutrition, and not having sufficient materials respectively were the greatest barriers to teaching nutrition in primary schools |
| Findholt  2016 [40]  Medium  USA | Survey | 1^0^ + M  87 | 87 | 87 | K-8 | NS | SCT | Intrapersonal  Interpersonal  School  Public Policy | Descriptives of classroom food practices, teacher eating behaviours and teacher reported beliefs about SFE and NK. 86% used candy as a reward, 42% used candy 2-3 times/month, 43% consumed SSB in classroom, >90% agreed nutrition important for learning and future health |
| Graham  2005 [41]  Poor  USA | Survey | 592 | 592 | NS | 4 | NS | NS | Intrapersonal  Interpersonal  School  External  Public Policy | Descriptive. Approximately half of the teachers strongly agreed that resources such as teacher training for gardening and its connection to curriculum (51%), curriculum materials linked to academic instruction (50%), and lessons on teaching nutrition in the garden (46%) would assist in the school garden being used for academic instruction. Greatest barriers to using the garden for academic instruction were time (67%), lack interest (63%), lack of experience (61%), lack of curricular materials linked to academic standards (60%), lack of knowledge (60%), and lack of teacher training in relation to gardening (58%). |
| Hamilton  2021 [42]  High  USA | Survey | 1^0^ + M + 2^0^  239 | 98 (41) | 188 (89) whole sample | K-12 | 13.6 (± 9.6)  whole sample | NS | Intrapersonal  School  Public Policy | Controlling for student SES, years of teaching experience (β = −.25, p < .001) and personal health (β = −.18, p = .02), were associated with using fewer unhealthy practices in the classroom. For healthy modelling (ate healthy food, drank water in the class), sig. predictors were teaching experience (β = −.31, p < .001), dieting (β = .32, p < .001), and personal health (β = .17, p = .02). |
| Hammersch-midt  2011 [43]  Poor  USA | Survey | 69 | 69 | NS | NS | NS | NS | Intrapersonal  School  External | The top 3 ways reported by respondents on how nutrition education was already integrated in their school, were implementing their school wellness policy (59%), a nutrition module within a comprehensive health curriculum (49%), and partnering with outside organizations/ individuals (48%). |
| Harris  2021 [44]  Low  USA | Survey | 130 | 130 | 106 (82) | K-6 | NS | NS | Intrapersonal  External | Descriptive: 64% had taught nutrition, of these 47% combined with other subjects;45% had taught up to 10 hrs in a year. 70% were interested in PD |
| Hart 2020 [45]  Low  USA  England | Survey | 1503 | 22% primary teachers | NS | Food teachers | NS | Sen’s Capability  Michie Behaviour Change | Intrapersonal  School  Public Policy | Budget, time, facilities, equipment and class size were perceived by senior school leaders as significant barriers to delivering high quality food teaching. Primary teachers described the following as fairly, very or a somewhat significant challenge: adequate budget – (86%), adequate time (88%), adequate facilities and equipment (78%), appropriate class size (69%). Less than 10 hrs was spent on food education by 56% of food teachers, and 63% of senior leaders. Whole school food policies were in only 30% of primary schools, 63% agreed that they regularly monitored compliance with school food standards. |
| Henry  2010 [46]  High  USA | Survey | 1^0^+M+2^0^ 345 | 99 (29) | 219 (77)  whole sample | K-12 | 57% >10 yrs | NS | Intrapersonal  Interpersonal  School External  Public Policy | Low scores re implementing NE teachers less agreement with items addressing their preparation to implement NE  Sex, grade level, and completion of a nutrition course sig predicted perceptions of NE Resources (F(7, 266) = 5.00, P < .01);  sex and completion of a nutrition course sig predicted School Food and Nutrition Guidelines (F(7, 266) = 2.37, P = .02); sex, grade level, completion of a nutrition course, and years of teaching experience sig predicted perceptions of Implementing Nutrition Education F(7, 266) = 9.89, P < .01; only grade level taught was the sig predictor of School Food Services F(7, 266) = 4.13, P < .01). |
| Jones  2015 [47]  High  USA | Survey | 100 | 51 (50) | 83 (83)  whole sample | Pre-K-12 | 57%>10 yrs  whole sample | NS | Intrapersonal  School | NK scores 5-53 mean 36/58  Predictors of NK were sex (p<.05), highschool teacher (p<.05) and Hispanic ethnicity (p<.01). Final model accounted for 41% of variance (F=15.01, p<.001) |
| Kinsler  2012 [48]  High  USA | Survey | 59 | Taught nutrition  59 | 48 (81) | 75% Grades 3-5 | 65% >11 yrs | NS | Intrapersonal | Mean NK 50% (16-100)  Mean SE 71%  Bivariate analyses: teachers with 11-15 years of experience were less likely than teachers with >16 years of experience to know how to read and interpret a food label (p<.05), teach students about reading food labels (p<.01) teach students about nutrition (p<0.05) |
| Lambert  2006 [49]  Poor  USA | Survey | 669 | 482 | NS | K-6 | 79% >10 yrs | NS | Intrapersonal  Interpersonal  School External  Public Policy | 66% of teachers provide 1-10 hrs of NE/school year, 69% of foodservice directors provide ≤1hr NE/school year. In response to statements, mean levels for teachers (2.9 ± 0.5) showed they agreed that nutrition education is valued at their schools. 93% of teachers agreed that NE should be part of the curriculum |
| Lambert  2010 [50]  High  USA | Survey | 321 | 221  Included | NS | Pre-K-6 | 45% >10 yrs | OCT | Intrapersonal  School  Public Policy | 58% believe they do not have adequate time for NE; 33% agreed in-service training was provided; 37% agreed resources were not provided for NE; 29% had opportunity to provide input into NE across curricula areas |
| Lambert 2016 [51]  Low  USA | Survey | 339 |  | 326 (96) | K-6 | 40%>10 yrs | TPB | Intrapersonal  Interpersonal | 93%, 80%, 70% indicated they allowed students to eat LNV foods in classroom for celebrations, rewards and incentives respectively (Behaviour). Attitude Toward the Behaviour (t = 4.04; P < .01), Subjective Norms (t = 3.78, P < .01), and Perceived Behavioural Control (t = 5.19; P < .01), were sig accounting for 32% of the variance. |
| Metos  2019 [52]  High  USA | Survey | 628 | 624 | 570 (91) | K-6 | 42% >10 yrs | SCT | Intrapersonal  Interpersonal  School  External | 97% agree nutrition impacts a child’s learning, 35% agree habits now will impact health as adults.  Mean Nutrition Impact score: 16.7 ± 5.0  68% agree they make a different to student eating, 52% confident to teach nutrition, 21% have support to teach nutrition, 70% responsibly to role model Mean SE score 13.6 ± 2.5 . Correlations between hrs of nutrition taught and teachers’ attitudes and beliefs (r = .37, p < .01), nutrition self-efficacy (r = .38, p < .01), and personal health practices (r = .15, p < .01) were weak, yet sig. |
| Perikkou 2015 [53]  High  Cyprus | Survey | 1436 | 1436 | 1189 (83) | NS | Pre-contemplation: 12.1 yrs  Contemplation: 11.9 yrs  Action: 14.5 yrs | TTM | Intrapersonal | More likely to be action phase for implementing NE: older teachers with a health specialisation (p<.001), those in good health and satisfied with own eating (p=.046), those with healthier eating habits, less likely to consider lack of time and resources or not as part of duties (p<.001)  More likely to action role modelling: older teachers (p=.001) with more experience (p=.006), with health specialisation (p=.018), female (p=.0033) and in good health (p=.016) those with healthier eating habits, less likely to consider lack of time and resources or not as part of duties (p<.001) |
| Prescott  2018 [54]  Medium  USA | Survey | 22 | 22 | 18 (90) | 4 | 10.7 ± 6.0 | NS | Intrapersonal | Teachers who reported they liked to cook (β = .53, P < .02) rated student engagement and academic skills higher, spending greater than 45 minutes preparing meals at home (β = .54, P < .01) were more comfortable providing assistance. Teacher BMI not related |
| Rafiroiu  2005 [55]  High  USA | Survey | 1^0^+M+2^0^ 99 | 40 | 36 (90) | K-5 | 90% > 5yrs | NS | Intrapersonal | NK mean 65%±1%  Nutrition attitudes mean 51.9±3.5  91% wanted more nutrition info and 93% nutrition should be taught in all grades |
| Rossiter  2007 [56]  High  USA | Survey | Pre-service  103 | Primary pre-service  52 | 75 (73)  whole sample | NA | NA | NS | Intrapersonal  Intrapersonal  School | 36% likely or very likely to allow students to eat candy in classroom  SFE score 10.0±4.4  PHI 10.1±2.1  NK 65%±18%  Higher classroom practice scores were associated with less support for the importance of a healthy school environment (p=.001), higher perception of personal health (p=.02) and being male (p=.035) |

^Paper quality, see Supplementary File 2.

*Elementary and primary school are used depending on country context

**Abbreviations**: 1^0^ + M + 2^0^ = primary/elementary + middle school + secondary/high school; B = baseline; C = control; CFIR = Consolidated Framework for Implementation Research; D&I = dissemination and implementation; F/u = follow-up; HBM = Health Belief Model; I = intervention; INEP = Integrated Nutrition Education Program; K = Kindergarten; LNV = Low nutritive value; NA = not applicable; NE = nutrition education; NK = nutrition knowledge; NS = not specified; OCT = Organisational Change Theory; PD = professional development; PE = physical education; PHI = personal health index; PRISM = Practical Implementation Sustainability Model; RE-AIM = Reach, Effectiveness, Adoption, Implementation, and Maintenance; SCT = Social Cognitive Theory; SE = Self Efficacy (Bandura); SEM = Socio-Ecological Model; SFE = school food environment; SSB = sugar sweetened beverages; TPB = Theory of Planned Behaviour; TTM = Transtheoretical Model (five stages of change).

**Supplementary File 3, Table 3**: Findings from the Mixed Method Paper

| **Author**  **Year**  **Quality^**  **Country** | **Method** | **Sample size**  **N** | **Participants**  **Elementary school teachers*** | | | | **Frameworks** | | **Main Findings** |
| --- | --- | --- | --- | --- | --- | --- | --- | --- | --- |
|  |  |  | **n (%)** | **Sex**  **F (%)** | **Grades** | **Years Teaching** | **Theoretical** | **Socio-ecological framework** |  |
| Bergling 2022 [68]  Cross-sectional: Poor  Qualitative: High  USA | Survey | 229 | 229 | 97 | K-5 | 22% >20 | RE-AIM, PRISM, CFIR | Intrapersonal  External | Mean effectiveness score was 16.6/24 (range 7-24); no adoption constructs were associated with program impact, relative priority (individual): teachers’ personal beliefs and values influenced their willingness to adopt INEP. Intervention-level factors interplayed with teacher perceptions. Teacher perception had a negative association with burden (β =-1.1, p=.05), i.e. the more difficult they found the program to implement the lower they would report its impact. Teacher perception had a positive association with workload (β =.95, p=.04), i.e. "the more time teachers reported having to dedicate to teaching program lessons, the higher they would report INEP impact" Quantitative analyses showed a negative association between the intervention-level factor of burden (β=−1.1, p=. 05) and a positive association with workload (β =. 95, p=. 04) with teacher perception. Block 2 Intervention and individual D&I factors burden/complexity (implement) (β = -1.2, p=.03), workload as individual factor .99 (.03). Block 3 Intervention and individual and organisation D&I factors burden/complexity (implement) (β = -1.3, p=.02), workload as individual factor (β = 1.0, p=.02) |

^Paper quality, see Supplementary File 2.

*Elementary and primary school are used depending on country context

**Abbreviations**: 1^0^ + M + 2^0^ = primary/elementary + middle school + secondary/high school; B = baseline; C = control; CFIR = Consolidated Framework for Implementation Research; D&I = dissemination and implementation; F/u = follow-up; HBM = Health Belief Model; I = intervention; INEP = Integrated Nutrition Education Program; K = Kindergarten; LNV = Low nutritive value; NA = not applicable; NE = nutrition education; NK = nutrition knowledge; NS = not specified; OCT = Organisational Change Theory; PD = professional development; PE = physical education; PHI = personal health index; PRISM = Practical Implementation Sustainability Model; RE-AIM = Reach, Effectiveness, Adoption, Implementation, and Maintenance; SCT = Social Cognitive Theory; SE = Self Efficacy (Bandura); SEM = Socio-Ecological Model; SFE = school food environment; SSB = sugar sweetened beverages; TPB = Theory of Planned Behaviour; TTM = Transtheoretical Model (five stages of change).

**Supplementary File 2, Table 4**: Findings from Qualitative Papers

| **Author**  **Year**  **Quality^**  **Country** | **Method** | **Sample size**  **N** | **Participants**  **Elementary school teachers*** | | | **Frameworks** | | **Main Findings** |
| --- | --- | --- | --- | --- | --- | --- | --- | --- |
|  |  |  | **Sex**  **F (%)** | **Grades** | **Years Teaching** | **Theoretical** | **Socio-ecological framework** |  |
| Aydin 2021^†^ [57]  Medium  Australia | Interview | 17 | 71 | NS | 30% >15 | Social Constructivism | Intrapersonal  Interpersonal  School | **Strengths**   - Food and nutrition education programs especially those with a practical component, have a positive impact on children - Being community based, primary schools were in a position to reach parents. - Teachers are recognised as important role models of healthy eating - Healthy school food environments affected food choices positively: a. Fruit breaks/ free fruit b. Rules over food breaks c. Healthy canteens and lunch orders   **Barriers to FNE**   - Limited time A lack of time, competing priorities overcrowded curriculum were regarded as a barrier. - Lack of knowledge, expertise and motivation, lack of teaching resources negatively impacts teacher confidence and knowledge - Unhealthy school food environments - Cost of food and nutrition education and lack of funding |
| Aydin 2022^†^ [58]  High  Australia | Interview | 17 | 71 | NS | 30% >15 | Social Constructivism | Intrapersonal  School  External | FNE was considered   - As important as other core subjects such as literacy and numeracy - Linked to acquiring important life skills impacting on current learning and current and future health status - A teacher/school responsibility - Needs to be regular and consistent - Primary school was considered good time to start as it gets harder as they get older   **Barriers to FNE**   - Time scarcity - Lack of PD - Lack of resources, school facilities and funding - Lack of staff to deliver practical classes - Lack of support from the principal |
| Beinert  2021 [59]  Medium  Norway | Focus group | 3 schools  80 teachers | 75 | Grades 2, 3, 6 | NS | NS | Intrapersonal  Interpersonal  School | - Feedback on specific program of activity Food & Health (FH) - Program was operationalised as cooking in practice: valued by students and teachers; teachers believed practical nature gave students opportunity to develop mastery in the subject; the practical aspect was especially valuable for academically weaker students. - Practical aspects were prioritised, teachers wanted to include nutrition education but had limited time - The focus on cooking and developing cooking skills was a missed opportunity for nutrition education. Nutrition education was delivered either before, during, or after cooking (while eating) or additional reading set (homework). The theory (nutrition education) and practice (cooking) were too detached. - Some schools reduced the time scheduled for the program - Resources need to: be adaptable/flexible, be user friendly, intuitive, have some leeway - a 'bank of ideas' available to pick up and adapt activities, would both help save time and be workable in their class - Focus in class on 'following the recipe' had less focus on elements like exploration, creativity, experimentation, which the FH curriculum says the subject should support. FH curriculum is more comprehensive, 'emphasising elements like critical thinking, sustainability and developing awareness of the connection between diet and health |
| Berggren  2021 [60]  Poor  Sweden | Web survey  responses to open-ended question | N=823 189 (23%) responded to question | NS | Grades 1-9 | NS | School lunch as social and pedagogical space after Lefebvre. | Intrapersonal  Interpersonal  School | - School lunch is an opportunity for social interaction with pupils; a meeting place, an informal setting suitable for meeting the pupils and for strengthening relationships. - prevention of bullying - not pedagogical but more for recreation and peer interaction. Opportunity to focus on social skills rather than formal pedagogy - School lunch was a challenging activity with teachers identifying the food served was not aligned with using it as a pedagogical tool (nutrition, environmental impact, hygiene etc), payment for the food and the overall working environment, and teachers’ rights and needs as employees. Participating in the school lunch created time pressure and stress and teachers identified the need to have downtime. |
| Bergling  2021 [61]  Medium  USA | Interview | 14 | NS | Grades K-5 | Most 5+ yrs with INEP | RE-AIM, PRISM, CFIR | Intrapersonal  School | Major barriers to implementation from the teacher perspective were ‘workload’ (the burden of implementing the intervention in addition to other responsibilities) and ‘time’ (the amount of time they had available to implement the intervention) were but as self-efficacy increased based on experience, the burden of implementation decreased. School culture: could act as enabler or barrier to INEP adoption. Intervention needs to be easy to use, simple and structured, easy to implement and fit in with curriculum, need the ability to pick and choose and not have to do all of it. |
| Gray  2016 [62]  High  USA | Focus group | 53 | 85 | NS | NS | HBM, SCT | Intrapersonal  Interpersonal  School | 1. Teachers believed that NE was important and they had a role in modelling health eating 2. School food environment was important with school meals potentially being the only meals some students received; snacks and junk food dominated children’s diets at school, home and in their neighbourhoods; teachers did not believe that children ate a balanced diet; junk food available in schools detracts from healthy eating. 3. The perceived quality of school meals was a barrier to healthy eating; negative feed-back sometimes received from school nutrition staff 4. Teachers believed that nutrition education, outreach efforts, changes in school meals and teacher modelling could help improve children’s eating habits |
| Hall  2016 [63]  High  USA | Interviews  Observation | 10 | NS | Grades K-2 | 2-12 yrs | Phenomenology | Intrapersonal  Interpersonal  School | 1. Teachers have meaningful roles wrt NE: educator, role model, coach; advocate, supporter, engager, guide, school “wellness champ,” and enlightener. Most roles were within the classroom; could extend to school-wide 2. NE was important compared to other school subjects, and formed the foundation of healthy lifestyle habits 3. Teachers influenced students' behaviour, knowledge and self-efficacy through the additional activities they offered and roles they played. Teachers influenced curriculum through adaptation for their individual classrooms The curriculum influenced students' behaviour, knowledge and self-efficacy through interactive qualities. It influenced teacher self-efficacy through simplicity and ease of use Students influenced teacher enjoyment of nutrition education through their positive attitude toward nutrition education 4. Barriers included time, prioritization of core subjects, resources, budget, and home environment |
| Koutsaki 2022 [64]  High  Greece | Interviews | 18 | 61 | 44%  K-6 | 8-30 yrs | NS | Intrapersonal  Interpersonal  School | NE should start from a young age, be compulsory in primary schools, no training, support, or additional education in nutrition, lack of educational resources. Teachers were influential and acted as role models. Dietary habits could be influenced by the relationship between the school and the parents - with students able to apply the knowledge they learn in school in the home environment. Most effective way of learning was experiential - and cooking was used as a learning approach. Identified barriers were: lack of knowledge and training leading to a lack of interest by teachers and principals, lack of time and the need to focus on other classes, NE not included in official curriculum and therefore had to be implemented after hours. |
| Maliotou 2022 [65]  Low  Cyprus | Interviews | 12 | NS | NS | NS | NS | Intrapersonal  School  External | Implementation is by enthusiastic teachers who are motivated. Curriculum focus makes it difficult to incorporate sustainable nutrition and its not clear what teachers would be responsible. Insufficient time to prepare and teach, focus on other units cannot deal with any depth. Economic and practical difficulties. |
| Prelip  2006 [66]  Poor  USA | Structured interview | 78 | 85 | NS | 75% ≥ 6 yrs | NS | Intrapersonal  School | Teachers: 46% teach nutrition, 28% model/encourage healthy eating, 19% my role is an important one, 14% lead, motivate, advocate and/or facilitate, 8% provide information sources, 8% teach parents nutrition, 5% provide healthy food. 85% defined NE as teaching or raising awareness about nutrition in students; 9% said that NE involves teaching parents and families; 89% reported integrating nutrition education into science; 85% into ESL; 83% into Health; 83% into Art; 72% into reading/language; teachers recognized a wide variety of resources for nutrition education within their schools, 8% of teachers reported that their school had no resources for nutrition education. Teachers believed that NE increases nutrition knowledge and interest in nutrition of students, 18% thought that NE affected food choices at home, 81% reported students eat better due to increased student awareness of 'healthy eating' (50%) and through making healthier food choices (25%). Time allocated to NE varied greatly: 28.2% >1hr/w, 20.5% 1-15min/w; 67.9% thought too little time was spent on NE. Reasons for why not more time is spent on NE: time 76%, other curricula 70%, inadequate teacher training 63%, inadequate school equipment 46% or facilities 37%. |
| Vio  2018 [67]  Poor  Chile | Focus group | 22 | 86 | Grades 3-5 | NS | NS | Intrapersonal  External | 1. Teachers on average rated the healthiness of their own eating habits as 4.4 (7 highest) eating habits were described as non-healthy and a complex issue that they deal with day to day, depending on the amount of time they have and their level of stress 2. Barriers to healthy eating included money, time, and motivation 3. Teachers on average rated their cooking skills as 5.3 (7 highest). Teachers agreed that they have the skills and potential to make healthy lunches. 4. When proposing an ICT teachers preferred a variety of games that were individualised 5. Teachers disagreed with ICT as an approach as they were trying to limit use of technology. Preference was for practical approaches such as cooking workshops, school gardens with vegetables, recycling, and other practical activities. |

^†^The data from these two papers was collected from the same cohort

**Abbreviations**: 1^0^ + M + 2^0^ = primary/elementary + middle school + secondary/high school; B = baseline; C = control; CFIR = Consolidated Framework for Implementation Research; D&I = dissemination and implementation; F/u = follow-up; HBM = Health Belief Model; I = intervention; INEP = Integrated Nutrition Education Program; K = Kindergarten; LNV = Low nutritive value; NA = not applicable; NE = nutrition education; NK = nutrition knowledge; NS = not specified; OCT = Organisational Change Theory; PD = professional development; PE = physical education; PHI = personal health index; PRISM = Practical Implementation Sustainability Model; RE-AIM = Reach, Effectiveness, Adoption, Implementation, and Maintenance; SCT = Social Cognitive Theory; SE = Self Efficacy (Bandura); SEM = Socio-Ecological Model; SFE = school food environment; SSB = sugar sweetened beverages; TPB = Theory of Planned Behaviour; TTM = Transtheoretical Model (five stages of change).
